# A Prospective Study of Plasma Vitamin D Metabolites, Vitamin D Receptor Polymorphisms, and Prostate Cancer

**DOI:** 10.1371/journal.pmed.0040103

**Published:** 2007-03-20

**Authors:** Haojie Li, Meir J Stampfer, J. Bruce W Hollis, Lorelei A Mucci, J. Michael Gaziano, David Hunter, Edward L Giovannucci, Jing Ma

**Affiliations:** 1 Channing Laboratory, Department of Medicine, Brigham and Women's Hospital, Harvard Medical School, Boston, Massachusetts, United States of America; 2 Department of Epidemiology, Harvard School of Public Health, Boston, Massachusetts, United States of America; 3 Department of Nutrition, Harvard School of Public Health, Boston, Massachusetts, United States of America; 4 Division of Preventive Medicine, Brigham and Women's Hospital, Harvard Medical School, Boston, Massachusetts, United States of America; 5 Massachusetts Veterans Epidemiology Research and Information Center, Veterans Administration Boston Healthcare System, Massachusetts, United States of America; 6 Department of Pediatrics, Medical University of South Carolina, Charleston, South Carolina, United States of America; McGill University, Canada

## Abstract

**Background:**

Vitamin D insufficiency is a common public health problem nationwide. Circulating 25-hydroxyvitamin D_3_ (25[OH]D), the most commonly used index of vitamin D status, is converted to the active hormone 1,25 dihydroxyvitamin D_3_ (1,25[OH]_2_D), which, operating through the vitamin D receptor (VDR), inhibits in vitro cell proliferation, induces differentiation and apoptosis, and may protect against prostate cancer. Despite intriguing results from laboratory studies, previous epidemiological studies showed inconsistent associations of circulating levels of 25(OH)D, 1,25(OH)_2_D, and several VDR polymorphisms with prostate cancer risk. Few studies have explored the joint association of circulating vitamin D levels with VDR polymorphisms.

**Methods and Findings:**

During 18 y of follow-up of 14,916 men initially free of diagnosed cancer, we identified 1,066 men with incident prostate cancer (including 496 with aggressive disease, defined as stage C or D, Gleason 7–10, metastatic, and fatal prostate cancer) and 1,618 cancer-free, age- and smoking-matched control participants in the Physicians' Health Study. We examined the associations of prediagnostic plasma levels of 25(OH)D and 1,25(OH)_2_D, individually and jointly, with total and aggressive disease, and explored whether relations between vitamin D metabolites and prostate cancer were modified by the functional *VDR* FokI polymorphism, using conditional logistic regression. Among these US physicians, the median plasma 25(OH)D levels were 25 ng/ml in the blood samples collected during the winter or spring and 32 ng/ml in samples collected during the summer or fall. Nearly 13% (summer/fall) to 36% (winter/spring) of the control participants were deficient in 25(OH)D (<20 ng/ml) and 51% (summer/fall) and 77% (winter/spring) had insufficient plasma 25(OH)D levels (<32 ng/ml). Plasma levels of 1,25(OH)_2_D did not vary by season. Men whose levels for both 25(OH)D and 1,25(OH)_2_D were below (versus above) the median had a significantly increased risk of aggressive prostate cancer (odds ratio [OR] = 2.1, 95% confidence interval [CI] 1.2–3.4), although the interaction between the two vitamin D metabolites was not statistically significant (*p*_interaction_ = 0.23). We observed a significant interaction between circulating 25(OH)D levels and the *VDR* FokI genotype (*p*_interaction_ < 0.05). Compared with those with plasma 25(OH)D levels above the median and with the FokI *FF or Ff* genotype, men who had low 25(OH)D levels and the less functional FokI *ff* genotype had increased risks of total (OR = 1.9, 95% CI 1.1–3.3) and aggressive prostate cancer (OR = 2.5, 95% CI 1.1–5.8). Among men with plasma 25(OH)D levels above the median, the *ff* genotype was no longer associated with risk. Conversely, among men with the *ff* genotype, high plasma 25(OH)D level (above versus below the median) was related to significant 60%∼70% lower risks of total and aggressive prostate cancer.

**Conclusions:**

Our data suggest that a large proportion of the US men had suboptimal vitamin D status (especially during the winter/spring season), and both 25(OH)D and 1,25(OH)_2_D may play an important role in preventing prostate cancer progression. Moreover, vitamin D status, measured by 25(OH)D in plasma, interacts with the *VDR* FokI polymorphism and modifies prostate cancer risk. Men with the less functional FokI *ff* genotype (14% in the European-descent population of this cohort) are more susceptible to this cancer in the presence of low 25(OH)D status.

## Abstract 2

Results of this study by Haojie Li and colleagues suggest that vitamin D deficiency is common among men in the US, and that vitamin D status and genetic variation in the*VDR* gene affect prostate cancer risk.

## Editors' Summary

**Background.:**

Prostate cancer occurs when cells in the prostate gland (part of the male reproductive system) accumulate genetic changes that allow them to grow into a disorganized mass of cells. Patients whose disease is diagnosed when these cells are still relatively normal can survive for many years, but for patients with aggressive cancers—ones containing fast-growing cells that can migrate around the body—the outlook is poor. Factors that increase prostate cancer risk include increasing age, having a family history of prostate cancer, and being African American. Also, there are hints that some environmental or dietary factors affect prostate cancer risk. One of these factors is vitamin D, of which high levels are found in seafood and dairy products, but which can also be made naturally by the body—more specifically, by sunlight-exposed skin. One reason researchers think vitamin D might protect against prostate cancer is that this cancer is more common in sun-starved northern countries (where people often have a vitamin D deficiency) than in sunny regions. Prostate cancer is also more common in African American men than in those of European descent (when exposed to the same amount of sunlight, individuals with darker skin make less vitamin D than those with lighter skin). Once in the human body, vitamin D is converted into the vitamin D metabolite 25-hydroxyvitamin D_3_ (25[OH]D) and then into the active hormone 1,25 dihydroxyvitamin D_3_ (1,25[OH]_2_D). This binds to vitamin D receptors (VDRs) and inhibits cell proliferation and migration.

**Why Was This Study Done?:**

The effect of 1,25(OH)_2_D on cells and the observation that related chemicals slow prostate cancer growth in rodents suggest that vitamin D protects against prostate cancer. But circulating levels of vitamin D metabolites in human male populations do not always reflect how many men develop prostate cancer. This lack of correlation may partly be because different forms of the VDR gene exist. One area of variation in the *VDR* gene is called the FokI polymorphism. Because everyone carries two copies of the *VDR* gene, individuals may have a FokI *FF,* FokI *Ff,* or FokI *ff* genotype. The *f* variant (or allele) codes for a receptor that is less responsive to 1,25(OH)_2_D than the receptor encoded by the FokI *F* allele. So levels of vitamin D sufficient to prevent cancer in one person may be insufficient in someone with a different FokI genotype. In this study, the researchers have investigated how levels of 25(OH)D and 1,25(OH)_2_D in combination with different *VDR* FokI alleles are influencing prostate cancer risk.

**What Did the Researchers Do and Find?:**

The researchers identified 1,066 men who developed prostate cancer between enrollment into the US Physicians' Health Study in 1982 and 2000, and 1,618 cancer-free men of the same ages and smoking levels as “controls.” They measured vitamin D metabolite levels in many of the blood samples taken from these men in 1982 and determined their FokI genotype. Two-thirds of the men had insufficient blood levels of vitamin D metabolites in the winter/spring; almost one-third had a vitamin D deficiency. Men whose blood levels of both metabolites were below average were twice as likely to develop aggressive prostate cancer as those in whom both levels were above average. Compared with men with high blood levels of 25(OH)D and the FokI *FF* or *Ff* genotype, men with low 25(OH)D levels and the FokI *ff* genotype were 2.5 times as likely to develop aggressive prostate cancer. However, men with the *ff* genotype were not at higher risk if they had sufficient 25(OH)D levels. Among men with the *ff* genotype, sufficient 25(OH)D levels might therefore protect against prostate cancer, especially against the clinically aggressive form.

**What Do These Findings Mean?:**

These findings confirm that many US men have suboptimal levels of circulating vitamin D. This vitamin is essential for healthy bones, so irrespective of its effects on prostate cancer, vitamin D supplements might improve overall health. In addition, this large and lengthy study reveals an association between low levels of the two vitamin D metabolites and aggressive prostate cancer that is consistent with vitamin D helping to prevent the progression of prostate cancer. It also indicates that the *VDR* FokI genotype modifies the prostate cancer risk associated with different blood levels of vitamin D. Together, these results suggest that improving vitamin D status through increased exposure to sun and vitamin D supplements might reduce prostate cancer risk, particularly in men with the FokI *ff* genotype. Because the study participants were mainly of European descent, the researchers caution that these results may not apply to other ethnic groups and note that further detailed studies are needed to understand fully how vitamin D affects prostate cancer risk across the population.

**Additional Information.:**

Please access these Web sites via the online version of this summary at http://dx.doi.org/10.1371/journal.pmed.0040103.

- MedlinePlus encyclopedia has pages on prostate cancer and on vitamin D
- Information for patients and physicians is available from the US National Cancer Institute on prostate cancer and on cancer prevention
- The Prostate Cancer Foundation's information on prostate cancer discusses the effects of nutrition on the disease
- Patient information on prostate cancer is available from Cancer Research UK
- Cancerbackup also has patient information on prostate cancer

## Introduction

Recent National Health and Nutrition Examination Survey (NHANES) data demonstrated that vitamin D insufficiency is a common public health problem nationwide, especially for elderly and minority populations [1]. A role for vitamin D in decreasing prostate cancer risk has been hypothesized on the basis of observations of higher prostate cancer mortality in regions of low solar radiation exposure and higher prostate cancer incidence in men of African descent, northern latitudes, and older age, all of which are associated with lower vitamin D status [2–4].

The prohormone vitamin D is obtained via UV exposure and from diet and supplements, and is hydroxylated by the liver to form 25-hydroxyvitamin D_3_ (25[OH]D). Circulating 25(OH)D is a sensitive marker of vitamin D status. Although the optimal range of 25(OH)D is debated [5], lower limits of 20 ng/ml (or 50 nM, to define deficiency) and 32 ng/ml (or 80 nM, to define suboptimal level or insufficiency) are favored by many researchers mainly based on functional indicators including parathyroid hormone, calcium absorption, and bone turnover markers [6–8]. In the kidney and other tissues, including prostate, 25(OH)D is further converted to the active hormone 1,25 dihydroxyvitamin D_3_ (1,25[OH]_2_D), which, operating through vitamin D receptors, inhibits cell proliferation, promotes angiogenesis and invasiveness, and induces differentiation and apoptosis [9–11]. Consistently, 1,25(OH)_2_D analogs slow prostate tumor growth in rodent models [12,13] and hinder prostate cancer metastasis [14], which indicates that 1,25(OH)_2_D may protect against both initiation and progression of cancer.

Despite the intriguing results from laboratory studies, epidemiological data from eight prospective studies showed inconsistent associations of prediagnostic circulating levels of vitamin D metabolites (25[OH]D and 1,25[OH]_2_D) with prostate cancer incidence [15–22]. Corder et al. [15] first reported an inverse association for circulating 1,25(OH)_2_D and aggressive prostate cancer, particularly in older men or in those with low 25(OH)D levels. Two studies of European Nordic countries measured only 25(OH)D; one found an inverse association [19], whereas the other found that both low and high 25(OH)D levels were associated with an increased risk [20]. All of the other five studies, including our earlier analysis from the present cohort [17], were conducted in the US and generally showed null or nonsignificant associations [16–18,21,22]. Of these five studies, three evaluated the joint association of 25(OH)D and 1,25(OH)_2_D with prostate cancer risk [17,18,22], and two (including our previous analysis) suggested that men with low levels of both metabolites had the highest risk [17,18]. However, none of these results were statistically significant, perhaps due to limited sample sizes, especially for patients with aggressive disease [17,18,22].

The vitamin D receptor (VDR), expressed in normal and malignant prostate cells, mediates the biological actions of 1,25(OH)_2_D [9,23–25]. Several common polymorphisms in the *VDR* gene have been described. A translation initiation codon polymorphism, the FokI restriction fragment length polymorphism (RFLP), identified recently [26,27], has no linkage disequilibrium with other *VDR* polymorphisms [28]. Although findings have been inconsistent [29–32], most studies indicate that the shorter *F* (versus *f*) allele is more responsive to 1,25(OH)_2_D [30] and has greater transcriptional activity [31,32]. The *F* allele has been associated with greater lumbar bone mineral density in several European-descent populations [27,33]. At the 3′ end of the *VDR* gene, a BsmI RFLP is strongly linked with several other polymorphisms, including ApaI, TaqI, and a poly-A repeat [32,34]. These polymorphisms produce no coding region differences and thus do not change the structure of the protein. A recent meta-analysis [35], which included 26 studies published through January 2005, showed overall no association between the FokI (eight studies) polymorphism or BsmI (ten studies) and risk of prostate cancer. The only study on the joint associations of circulating vitamin D levels and the *VDR* BsmI polymorphism with prostate cancer came from our group [36], showing that the BsmI *BB* genotype was associated with lower risk among men with low 25(OH)D status. Two recent case-control studies reported that the *ff* genotype was associated with increased risk of prostate cancer only in the presence of high sun exposure [37,38]. To date, no study has assessed the interaction of circulating vitamin D levels with the functional FokI polymorphism.

We therefore conducted a nested case-control study within the Physicians' Health Study (PHS), with nearly twice the sample size to extend the previous analyses [17,36]. With prostate cancer patients diagnosed between 1982 and 2000 (i.e., before and after prostate-specific antigen [PSA] screening became widespread), we specifically tested the following hypotheses: (1) lower plasma levels of 25(OH)D, 1,25(OH)_2_D, or both metabolites are associated with increased risk of prostate cancer, and the association is more pronounced for aggressive disease and among older men; and (2) men who carry the functional FokI *f* allele (which is less responsive to vitamin D signaling) have higher risk, especially if they have low vitamin D status. We also extended the previous analysis [36] of the BsmI polymorphism with prostate cancer and to study their possible interactions with vitamin D metabolites.

## Methods

### Study Population

The PHS was a randomized, double-blind, placebo-controlled trial of aspirin and β-carotene among 22,071 healthy US male physicians, aged 40–84 y, that began in 1982 [39]. The aspirin arm was terminated at the end of the fifth year due to a reduction in the risk of myocardial infarction; the β-carotene component of the trial continued until 1995, and the men are still followed. Written consent was obtained from each participant, and the investigation was approved by the Human Subjects Committee at Brigham and Women's Hospital. Men were excluded at baseline if they had a history of myocardial infarction, stroke, transient ischemic attack, or unstable angina; cancer (except for nonmelanoma skin cancer); current renal or liver disease, peptic ulcer, or gout; or current use of platelet-active agents, vitamin A, or β-carotene supplements. Study participants provided baseline information (including lifestyle habits such as dairy food intake and vigorous physical activity) via self-administrated questionnaires. Before randomization, 14,916 men (68%) provided a blood sample [17], and more than 70% of the specimens were received between September and November in 1982. Additional questionnaires were mailed at 6 months, 12 months, and annually thereafter to obtain medical information. Study investigators, unaware of the questionnaire or assay data, verified the reports of prostate cancer by participants and reviewed medical records and pathological reports to determine the tumor Gleason score, grade, and stage, according to the modified Whitmore-Jewett classification scheme [40]. Patients without pathologic staging were classified as indeterminate stage unless there was clinical evidence of distant metastases at diagnosis. Through 2000, follow-up was over 99% complete; vital status was ascertained for 100% of the participants by 2004.

Prostate cancer patients for the current study were drawn from participants who provided blood specimens at baseline (i.e., from both the treatment and placebo arms). For each patient, we selected one to three control participants at random from those who had provided blood, had not had a prostatectomy, and had not reported a diagnosis of prostate cancer at the time the diagnosis was reported by the case participant. Control participants were individually matched to case paricipants by age (±1 y and ±5 y for older men) and smoking status (never, former, or current).

### Laboratory Assessment for Vitamin D Metabolites and VDR Polymorphisms

Plasma concentrations of 25(OH)D and 1,25(OH)_2_D were determined by radioimmunosorbant assay in two separate batches (226 patients and their matched control participants in 1994, and 266 patients and their matched control participants in 2003) in the laboratory of Dr. Bruce Hollis (Medical University of South Carolina, Charleston, South Carolina, United States) as described previously [41,42]. Samples for each patient and matched control participant(s) were analyzed together, but in random order, with the patient status unknown to the laboratory personnel. The mean intra-pair coefficients of variation for blinded duplicate quality control samples were 7.9% to 8.1% for 25(OH)D and 1,25(OH)_2_D for the two batches.

DNA was extracted from baseline blood specimens for these patients and controls. With the laboratory personnel blinded to patient–control status, the FokI and BsmI genotypes were analyzed at the Dana Farber/Harvard Cancer Center Genotyping Core. The *VDR* RFLP genotypes were determined by PCR amplification, followed by restriction enzyme digestion, as described previously [27,43].

### Statistical Analysis

Of men who provided blood samples at the baseline, we studied 1,066 men who developed prostate cancer during 1982–2000 (18 y of follow-up) and 1,618 matched control participants. Of these, limitations in funding permitted measuring baseline plasma vitamin D concentrations (25[OH]D and 1,25[OH]_2_D) for 492 patients with prostate cancer (diagnosed between 1982 and 1995) and 664 matched control participants. For VDR polymorphisms, 1,034 patients (and 1,566 control participants) had the FokI genotype data, and 1,010 patients (and 1,432 control participants) had the BsmI genotype data. We then had 461 to 471 patients and matched control participants to evaluate the potential interactions between plasma vitamin D metabolites and the VDR polymorphisms in relation to prostate cancer risk.

We compared allele and genotype frequencies between patients and control participants using the χ^2^ test. Because plasma vitamin D levels were not normally distributed, we selected nonparametric techniques for the comparisons of vitamin D metabolites between patients and control participants for batches one and two, separately. Since the results for all the following analyses were similar between batches, we combined data from the two batches and performed matched analyses. We examined the association of circulating levels of 25(OH)D and 1,25(OH)_2_D in quartiles (with the highest quartile, defined by the distribution among controls, as the reference) and polymorphisms of FokI and BsmI (with the wild-type genotype as the reference) with risk of total prostate cancer. We then separately performed subgroup analyses for various subtypes of prostate cancer according to disease stage and grade at diagnosis, and whether those men developed metastases or died from prostate cancer by 2004. Because “high-grade” (Gleason score of 7–10) and “high-stage” (stage C or D) are the strongest predictors of prostate cancer death, the associations (both direction and magnitude) of these subgroup analyses were similar to those of “metastatic/fatal” cancers, and the sample size for each cancer subtype was relatively small, we reported the results for aggressive prostate cancer, when these subgroups were combined, and for nonaggressive disease, those diagnosed with stage A or B and low-grade (Gleason score of 2–6) prostate cancer. We further conducted stratified analyses by duration of follow-up, age at diagnosis (younger than 65 y or 65 y and older) or median age at baseline, and assessed age as a potential effect modifier. Because the levels of 25(OH)D (but not 1,25[OH]_2_D) were higher in samples collected during summer/fall and were lower in those collected during winter/spring (Table 1), presumably due to the difference in sun exposure, we used season- and batch-specific cutoff points for 25(OH)D and batch-specific cutoff points for 1,25(OH)_2_D based on levels of the control subjects. We then examined the joint associations of 25(OH)D with 1,25(OH)_2_D with the dichotomized variables (i.e., below or above median levels). Furthermore, we explored the interactions between these vitamin D metabolites and VDR polymorphisms in modifying prostate cancer risk. For VDR polymorphisms, we compared men who carried the homozygous variant genotype with the rest (the wild-type and the heterozygous genotypes combined) as the reference group. To examine whether any associations between vitamin D status and risk of prostate cancer were due to an effect of latent disease on plasma vitamin D status, we repeated all these analyses by excluding the patients diagnosed in the first 2 y of follow-up after the blood collection or the patients with baseline PSA levels 4 ng/ml or higher. Given that the PHS was a randomized trial, we also tested whether the associations of vitamin D, VDR polymorphisms with prostate cancer were modified by the β-carotene and the aspirin treatments.

**Table 1:**
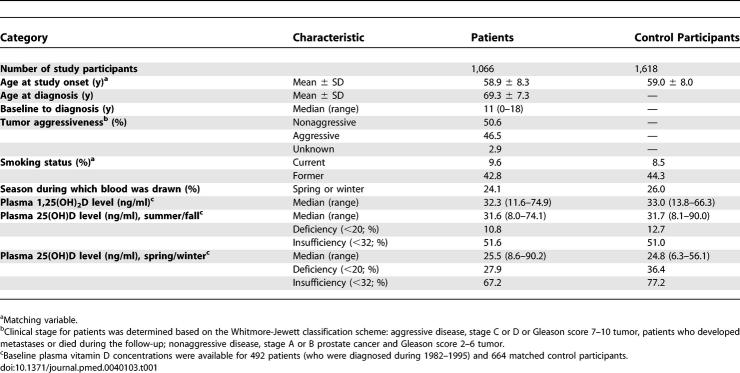
Baseline Characteristics of Patients with Prostate Cancer and Control Participants: The PHS

Odds ratios (ORs) and 95% confidence intervals (CIs) were calculated using conditional logistic regression models. Besides controlling for age, smoking, and follow-up period via the matched analysis, we adjusted for exercise (sufficient to induce sweating <1, 1–4, or ≥5 times per week) and race (European descent, yes or no). Because only 6% of the men in this study were not of European descent, we also repeated all the analyses excluding these individuals. We presented models without adjusting for dairy food (calcium) intake because adjustment for this factor did not change the results. For men (15 patients and 10 control participants) with missing information for exercise, we assigned them into the median category (i.e., 1–4 times/wk) and retained them in the analyses; including or excluding these men provided similar results. Tests for trend for the vitamin D metabolites were conducted by use of median levels of quartiles. To test for interactions, we analyzed models with and without the cross-product term with the two main exposures as continuous variables and conducted the likelihood ratio test. All statistics were calculated by SAS (version 8.12; SAS Institute Inc, http://www.sas.com) with a two-sided significance level of 0.05. We tested for heterogeneity for the associations with aggressive versus nonaggressive disease using the Stata Statistical Software, version 9.0 (http://www.stata.com).

## Results

All baseline characteristics presented in Table 1 were similar between patients with prostate cancer and control participants. Of the 1,066 incident patients, 496 had aggressive disease, 539 had nonaggressive prostate cancer, and 31 were unable to be classified because of insufficient information. The median interval from baseline in 1982 to diagnosis was 11 y, and the average follow-up duration after diagnosis was 9 y.

Approximately 75% of the blood samples were collected during summer/fall, among which levels of 25(OH)D and 1,25(OH)_2_D were significantly and positively correlated (*n* = 480 control participantss, Spearman correlation *r* = 0.17, *p* < 0.001); however, they were unrelated among samples collected during winter/spring (*n* = 184 control participants). Circulating levels of 1,25(OH)_2_D are physiologically tightly controlled and thus did not vary by season. Plasma 25(OH)D levels were higher in the “summer/fall” samples (median = 31.7 ng/ml among control participants) than in the “winter/spring” samples (median = 24.8 ng/ml). We categorized men with plasma 25(OH)D levels <20 ng/ml as vitamin D “deficient” and <32 ng/ml as “insufficient” [6–8]. Among control participants, the prevalence of vitamin D deficiency was 12.7% for blood samples collected in the “summer/fall” and 36.4% for the samples collected in “winter/spring” (Table 1); nearly 50% (summer/fall) to over two-thirds (winter/spring) of the men had insufficient vitamin D. Men not of European descent had an even higher prevalence of vitamin D insufficiency. The prevalence of deficiency was 16.7% in the “summer/fall” (*n* = 30) and 46.2% in “winter/spring” (*n* = 13) samples; the corresponding prevalent rates of vitamin D insufficiency were 63.3% and 92.3%. The overall vitamin D status of the participants in this cohort was similar to several other studies [16–18,21,22] as well as to US men in NHANES [1]. In contrast, the 25(OH)D levels were much lower (median ≤20 ng/ml) among men in the study by Corder et al. [15] and in two Nordic studies [19,20].

Levels of 25(OH)D and 1,25(OH)_2_D alone were not associated with risk of total or nonaggressive prostate cancer. Lower levels of 1,25(OH)_2_D tended to be associated with increased risk of aggressive prostate cancer (*p*_heterogeneity_, aggressive versus nonaggressive disease = 0.02), especially among men aged of 65+ y at diagnosis (*p*_trend_ = 0.03, Table 2); however, the interaction of vitamin D status with age, categorized by median or into four groups (<60, 60–65, 65–70, and 70+ y), or as a continuous variable, was not significant. In this cohort, the FokI and BsmI genotype frequencies were similar to previously reported populations of European descent [36,44–47], and the distributions were in Hardy-Weinberg equilibrium. We observed no direct relationship of the VDR FokI or BsmI polymorphism with risk of total, nonaggressive, and aggressive prostate cancer (Table 3), and the associations did not differ by age at diagnosis.

**Table 2:**
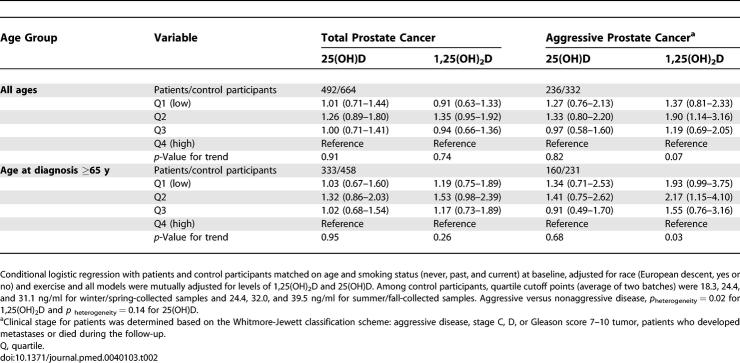
OR and 95% CI for Total and Aggressive Prostate Cancer According to Quartile Levels of Baseline Vitamin D Metabolites

**Table 3:**
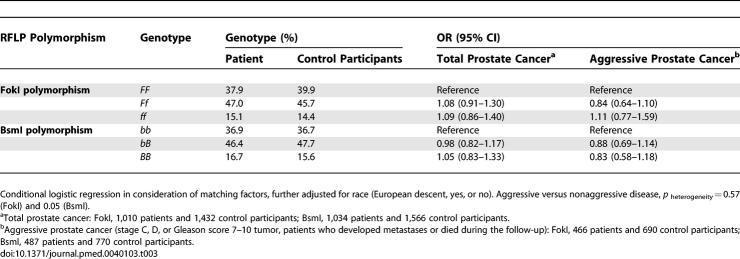
Association Between *VDR* Gene Polymorphisms and Risk of Total and Aggressive Prostate Cancer

We further extended our previous analysis [17,36] to evaluate the joint association of 25(OH)D and 1,25(OH)_2_D. Compared with men whose levels of both metabolites were above the median, men with circulating 25(OH)D (<32.0 ng/ml for “summer/fall” samples and <24.4 ng/ml for “winter/spring” samples; i.e., insufficient or deficient) and 1,25(OH)_2_D below the median had significantly increased risk of aggressive prostate cancer (OR = 2.06, 95% CI 1.24–3.43; *p*_heterogeneity_, aggressive versus nonaggressive disease = 0.01; Table 4), though the interaction between the two metabolites was not significant. Among men aged 65+ y at diagnosis, the association was slightly stronger (OR = 2.47, 95% CI 1.29–4.75), but formal tests for interactions between the vitamin D metabolites and age were not statistically significant. The associations were similar regardless of season of blood collection. Similar association was also observed for the risk of fatal/metastatic prostate cancer with the corresponding OR of 1.63 (95% CI 0.73–3.65). The 95% CI was wide probably due to small sample size (98 patients and 144 control participants).

**Table 4:**
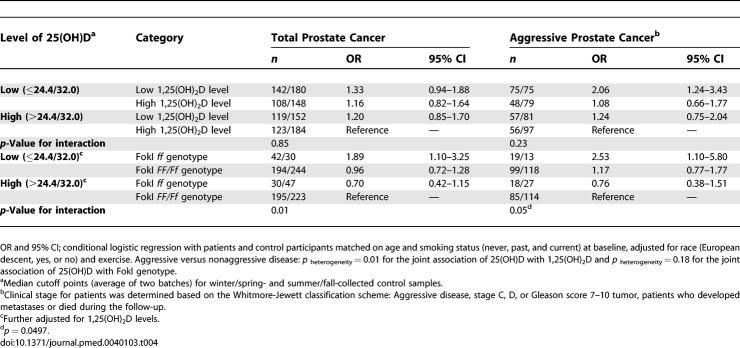
Joint Association of Plasma Level of 25(OH)D with 1,25(OH)_2_D or with Vitamin D Receptor Gene Polymorphisms in Relation to Total and Aggressive Prostate Cancer

We found significant interactions between circulating 25(OH)D (but not 1,25[OH]_2_D) levels and the FokI polymorphism in modifying prostate cancer risk (Table 4). Compared with those with plasma 25(OH)D levels above the median and with the FokI *FF or Ff* genotype, men who had low (or suboptimal) 25(OH)D levels and the less functional FokI *ff* genotype had 2-fold increased risks of total (OR = 1.89, 95% CI 1.10–3.25) and aggressive prostate cancer (OR = 2.53, 95% CI 1.10–5.80; OR for fatal/metastatic disease = 2.40, 95% CI 0.69–8.38), with a nonsignificant *p*_heterogeneity_ (aggressive versus nonaggressive disease) of 0.18. The FokI *ff* genotype (versus *FF/Ff*) was associated with increased risk of total (OR = 1.97, 95% CI 1.15–3.35; *p*_interaction_ = 0.01) and aggressive prostate cancer (OR = 2.16, 95% CI 0.97–4.82, *p*_interaction_ = 0.05; OR for fatal/metastatic disease = 1.83, 95% CI 0.55–6.11, *p*_interaction_ = 0.43) only among men with low 25(OH)D status but not among those with plasma 25(OH)D levels above the median. Conversely, among men who carried the FokI *ff* (but not *FF/Ff*) genotype, high (versus low) 25(OH)D was associated with reduced risk for total (OR = 0.37, 95% CI 0.18–0.74) and aggressive prostate cancer (OR = 0.30, 95% CI 0.11–0.82; OR for fatal/metastatic disease = 0.26, 95% CI 0.06–1.22). Our extended analyses showed no overall interactions of the BsmI polymorphism with vitamin D metabolites.

Although inverse associations between vitamin D levels and risk of developing aggressive disease were stronger for those diagnosed in the pre-PSA era, the trends were similar for both pre- and post-PSA era patients. Excluding patients diagnosed during the first 2 y of follow-up after blood collection or those who had baseline PSA levels ≥ 4 ng/ml did not significantly alter the relationship. We found similar associations between plasma vitamin D metabolites, VDR polymorphisms, and prostate cancer with (as presented above) and without (unpublished data) adjusting for race. The relations between VDR polymorphism and prostate cancer remained the same, and the inverse associations of plasma vitamin D levels with risk of prostate cancer were attenuated but remained significant, when the men of non-European descent were excluded. Men with circulating levels of both 25(OH)D and 1,25(OH)_2_D below (versus above) the median had a 1.7-fold (95% CI 1.03–2.95; versus 2.1-fold among all men; Table 4) increased risk of aggressive prostate cancer among men of European descent; these associations were stronger among those not of European descent (unpublished data), probably because of their low 25(OH)D status as described above. We found no interactions of vitamin D level or the VDR polymorphisms with the β-carotene and aspirin treatments in modifying prostate cancer risk.

## Discussion

In this large prospective cohort of middle-aged US male physicians, almost one-third of the men had vitamin D deficiency (25[OH]D <20 ng/ml), and more than two-thirds had insufficient vitamin D status (25[OH]D <32 ng/ml) in the winter/spring. Even in the summer/fall, more than 10% were vitamin D deficient, and more than half had insufficient vitamin D status (Table 1). These findings, consistent with most observations from other studies [16–22] as well as the recent NHANES [1], suggest an alarming problem of low vitamin D status in the US and in Northern European countries.

Prediagnostic 1,25(OH)_2_D levels tended to be inversely associated with risk of aggressive prostate cancer, especially among men aged 65+ y at diagnosis (Table 2) or among men with low levels of 25(OH)D (Table 4). Our findings were consistent with Corder et al., who first reported an inverse association for circulating 1,25(OH)_2_D and aggressive prostate cancer, particularly in older men, although the lowest risk was observed among men with high 1,25(OH)_2_D but low 25(OH)D levels [15]. Normura et al. found that men with low levels of both metabolites had the greatest risks (not statistically significant); however, they did not distinguish aggressive from nonaggressive cancer [18]. Ahonen et al. found an inverse association of 25(OH)D with prostate cancer in Finland [19], and Tuohimaa et al. found a U-shaped relationship between 25(OH)D and prostate cancer [20], but none of these two studies measured 1,25(OH)_2_D levels. Other prospective studies generally found no associations [16,17,21,22]. One major factor that may contribute to these inconsistent findings is that most studies did not specifically examine aggressive prostate cancer, the etiology of which appears to differ from that of indolent disease [16,17,21,22]. Another related factor may be the apparent differences in vitamin D status in various populations (Table 5) [15–22]. The overall vitamin D status of the participants was fairly low in the three studies showing significant inverse association with 1,25(OH)_2_D [15] or 25(OH)D levels [19,20]. The median levels of 25(OH)D for men of these studies were around or below 20 ng/ml so that at least half of the study participants were vitamin D deficient. In contrast, among the studies that did not find a direct association between circulating vitamin D metabolites and prostate cancer risk (including ours), the median levels of 25(OH)D (all seasons combined) were 29 ng/ml or higher, and the prevalence of vitamin D deficiency was approximately 20% [16–18,21,22]. Thus, men in Hawaii [18] and Baltimore [16] may have more sun exposure compared to those in Nordic countries [19,20], and the physicians [17] and health professionals [22] may be more conscious about nutrition and consume more supplements than the general population.

**Table 5:**
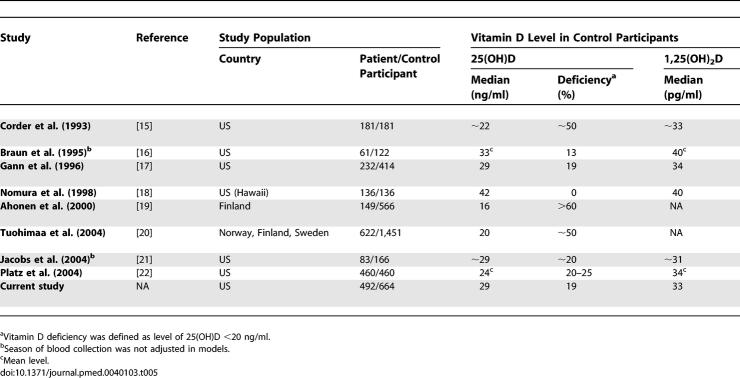
Prospective Studies of Circulating Level of Vitamin D Metabolites and Prostate Cancer Risk

**Table 5:**
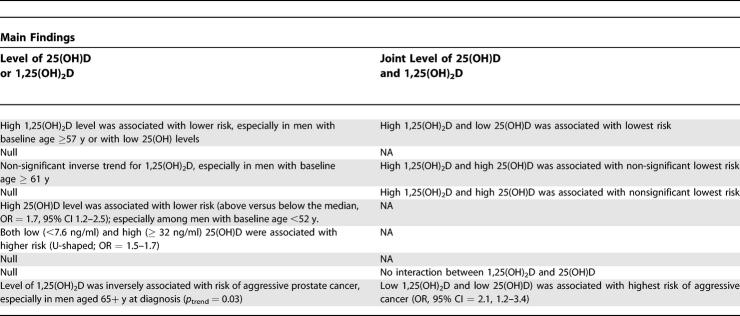
Extended.

We defined low 25(OH)D status as below the median (i.e., <24.4 ng/ml), close to deficient for blood samples collected in winter/spring, and <32.0 ng/ml, close to insufficient levels for blood samples collected in summer/fall. In our study, men with low levels of both 25(OH)D and 1,25(OH)_2_D, which may be a true indication of vitamin D deficiency, were at significantly increased risk for aggressive prostate cancer. Although with few men of non-European descent in this cohort, the data suggested stronger inverse associations between plasma vitamin D levels and risk of prostate cancer among them (versus men of European descent), probably because these men had poorer 25(OH)D status related to their darker skin color.

A significant association of plasma 1,25(OH)_2_D levels and risk of aggressive prostate cancer was apparent only among older men or men with insufficient 25(OH)D status, suggesting a role of 1α-hydroxylase activity in prostate cancer development and progression. Levels of the active hormone 1,25(OH)_2_D could be influenced by 25(OH)D status as well as 1α-hydroxylase activity. With low 25(OH)D status, 1,25(OH)_2_D levels could be maintained by increased 1α-hydroxylase activity, possibly explaining why we observed no correlation between the two metabolites in blood samples collected in winter/spring. Reduced enzyme activity of 1α-hydroxylase due to aging [48] or other factors, especially under low 25(OH)D status, could predispose a man to a higher risk of prostate cancer as observed in our study. This notion is indirectly supported by two studies that recently showed profoundly reduced 1α-hydroxylase activity in prostate cancer cell lines compared with cells from normal tissues [49,50], suggesting that these cells may have reduced or lost the ability to convert 25(OH)D to 1,25(OH)_2_D locally [51]. Because circulating 1,25(OH)_2_D level is relatively stable, an alternative explanation is that low 1,25(OH)_2_D, in concert with low 25D, may act as a better marker of low vitamin D status.

Neither the FokI nor the BsmI polymorphism was directly associated with prostate cancer in our study, which is consistent with previous observations [35,37]. However, we found an increased risk of prostate cancer associated with the less functional FokI *ff* genotype only in the presence of low 25(OH)D status. Most previous studies, summarized by Berndt et al. [35], were small and studied primarily localized disease. However, two studies reported an increased risk of prostate cancer associated with the FokI *ff* genotype was found in the presence of high sun exposure (thus, presumably higher 25[OH]D status) [37,38]. More studies are needed to resolve these apparently contradictory findings.

The strengths of this study include a prospective design with up to 18 y of follow-up and careful collection and storage of blood specimens and thorough ascertainment of events. Our large sample size, especially for patients with clinical aggressive prostate cancer, allowed us to assess the associations of 25(OH)D and 1,25(OH)_2_D, individually and jointly, with total and aggressive disease, as well as their potential interactions with the VDR polymorphisms. One limitation is that vitamin D levels were assessed in plasma collected at one time point and measured in two batches. However, the reproducibility of these assays was good as indicated by the low mean intra-pair coefficients of variation (both were <10%). Furthermore, the mean levels and their distribution were similar to those reported using fresh samples, and the overall- and batch specific-correlations between 25(OH)D with age and seasons of the year were as expected, supporting the internal validity of these assays. To ensure the comparability between patients and control participants and to reduce the nondifferential measurement errors due to batch-to-batch variation, patients and control participants were assayed together and analyzed in matched pairs, and we used batch-specific cutoff points to define the categories and utilized conditional logistic regression models for all the analyses. Nevertheless, if any such nondifferential measurement error exists, we expect that the strength of the association could be diluted toward the null. Other limitations included the lack of information on family history of prostate cancer and PSA screening practice, as well as PSA levels at diagnosis for these men. Findings in this cohort of physicians of predominantly European descent may not be easily generalized to other ethnic groups. Studies of other ethnic groups are necessary to better understand the role of vitamin D on prostate cancer.

In summary, the inverse association of 1,25(OH)_2_D alone or together with 25(OH)D with aggressive prostate cancer provide further evidence that both 25(OH)D and 1,25(OH)_2_D may play an important role in preventing prostate cancer progression, especially among older men. The FokI polymorphism may interact with 25(OH)D and modify prostate cancer risk. Men with the FokI *ff* genotype (14% in the European-descent population of this cohort) are more susceptible to this disease in the presence of low 25(OH)D status. Vitamin D insufficiency is a common problem, and improving vitamin D status through moderate sun exposure and vitamin D supplements, in particular, is essential for optimal health.

## Supporting Information

Alternative Language Abstract S1 Translation of the Abstract into Chinese by Haojie Li (24 KB DOC)

## Abbreviations

1,25(OH)_2_D: 1,25 dihydroxyvitamin D_3_
25(OH)D: 25-hydroxyvitamin D_3_
CI: confidence interval
NHANES: National Health and Nutrition Examination Survey
OR: odds ratio
PSA: prostate-specific antigen
PHS: Physicians' Health Study
RFLP: restriction fragment length polymorphism
VDR: vitamin D receptor
