# Supplementary material for: A Prospective Study of Plasma Vitamin D Metabolites, Vitamin D Receptor Polymorphisms, and Prostate Cancer
Source: PLoS Med. 2007 Mar 20;4(3):e103. doi: 10.1371/journal.pmed.0040103 (PMC1831738; doi:10.1371/journal.pmed.0040103)
Supplement: Alternative Language Abstract S1 — (24 KB DOC) [file pmed.0040103.sd001.doc]

**血浆中维生素D_3_代谢物水平及维生素D受体的多态性**

**与前列腺癌相关性的前瞻性研究**

**[摘要]**

**前言：**

维生素D缺乏是一个在全国范围内普遍存在的公共卫生问题。血浆中25-羟维生素D_3_（25(OH)D）是评价机体维生素D缺乏的常用指标，它在体内转化为具有活性的 1,25-二羟维生素D_3_（1,25(OH)_2_D）。1,25(OH)_2_D作用于维生素D受体（VDR）后可抑制体外细胞的增殖，诱导细胞分化和凋亡，因此有抗前列腺癌的保护作用。有关这方面的实验室研究结果很吸引人，但有关人体中维生素D_3_代谢物的水平，维生素D受体的多态性与前列腺癌相关性的流行病学研究，其结果是不一致的，且绝大部分研究未对维生素D_3_代谢物的水平和其受体的多态性间的相互关系进行探讨。

**方法和结果：**

**经**对“Physicians’Health Study”中14916名从未患有癌症的健康男性进行了18年的随访，以其中被确诊患前列腺癌1066人（其中包括496名分期为C或D、GLEASON评分为7-10、有局部及其它器官转移的致死性前列腺癌患者）为研究对象；再以在同一人群中经年龄和吸烟状况配对的1618名未患癌症者为对照；用条件逻辑回归模型，对这两组人群 癌症发病前的血浆25(OH)D和1,25(OH)_2_D水平（单独或一起）与前列腺癌和恶性前列腺癌发病的相关性进行了分析，并对其相关性是否受维生素D受体多态性的影响进行了分析。

结果看到研究对象冬春季血浆25(OH)D的中位数为25 ng/mL，夏秋季血浆水平的中位数为32 ng/mL。在对照人群中有13％（夏秋季）和36％（冬春季）的人其血浆25(OH)D水平＜20 ng/mL，为25(OH)D缺乏；有51％（夏秋季）和77％（冬春季）的人其血浆25(OH)D＜32 ng/mL，即低于理想水平。血浆1,25(OH)_2_D 水平不随季节变换而变化。

与血浆25(OH)D和1,25(OH)_2_D水平均高于中位数者相比，水平均低于中位数者发生恶性前列腺癌的危险性高（比值比 OR = 2.1，95%可信区间为1.2-3.4）；而且，25(OH)D和1,25(OH)_2_D之间没有相互作用. 我们还观察到体内25(OH)D水平和维生素D *Fok*I受体的多态性之间有相互作用（P<0.05）。 与25(OH)D水平高于中位数，并携带*Fok*I *FF* or *Ff*基因型者相比, 25(OH)D水平低于中位数并携带低效的*Fok*I *ff*基因型者发生前列腺癌（比值比 OR = 1.9, 95%可信区间为1.1-3.3）及恶性前列腺癌的危险性高（比值比 OR = 2.5，95%可信区间为1.1-5.8）。 在25(OH)D水平高于中位数的人群中, *Fok*I *ff*基因型和前列腺癌的发病没有相关性. 然而, 在携带*Fok*I *ff*基因型的人群中, 25(OH)D水平高（相对与水平低）者发生前列腺癌及恶性前列腺癌的危险性减低60-70%。

**结论:**

研究结果显示有相当大的比例的美国男性维生素D的水平低于理想水平(特别是在冬春季节)。25(OH)D和1,25(OH)_2_D在防止前列腺癌的进展起重要作用，且血浆25(OH)D水平和维生素D*Fok*I受体的多态性之间有相互作用。体内25(OH)D水平低，又携带*Fok*I *ff*基因型的男性（我们的研究人群中有14%的白人男性携带该基因型）对前列腺癌易感。
